# AsmMix: an efficient haplotype-resolved hybrid *de novo* genome assembling pipeline

**DOI:** 10.3389/fgene.2024.1421565

**Published:** 2024-07-26

**Authors:** Chao Liu, Pei Wu, Xue Wu, Xia Zhao, Fang Chen, Xiaofang Cheng, Hongmei Zhu, Ou Wang, Mengyang Xu

**Affiliations:** ^1^ BGI, Tianjin, China; ^2^ BGI Research, Shenzhen, China; ^3^ MGI Tech, Shenzhen, China; ^4^ BGI Research, Qingdao, China

**Keywords:** long reads, bioinformatics, *de novo*, genome assembly, haplotype, hybrid

## Abstract

Accurate haplotyping facilitates distinguishing allele-specific expression, identifying cis-regulatory elements, and characterizing genomic variations, which enables more precise investigations into the relationship between genotype and phenotype. Recent advances in third-generation single-molecule long read and synthetic co-barcoded read sequencing techniques have harnessed long-range information to simplify the assembly graph and improve assembly genomic sequence. However, it remains methodologically challenging to reconstruct the complete haplotypes due to high sequencing error rates of long reads and limited capturing efficiency of co-barcoded reads. We here present a pipeline, AsmMix, for generating both contiguous and accurate diploid genomes. It first assembles co-barcoded reads to generate accurate haplotype-resolved assemblies that may contain many gaps, while the long-read assembly is contiguous but susceptible to errors. Then two assembly sets are integrated into haplotype-resolved assemblies with reduced misassembles. Through extensive evaluation on multiple synthetic datasets, AsmMix consistently demonstrates high precision and recall rates for haplotyping across diverse sequencing platforms, coverage depths, read lengths, and read accuracies, significantly outperforming other existing tools in the field. Furthermore, we validate the effectiveness of our pipeline using a human whole genome dataset (HG002), and produce highly contiguous, accurate, and haplotype-resolved assemblies. These assemblies are evaluated using the GIAB benchmarks, confirming the accuracy of variant calling. Our results demonstrate that AsmMix offers a straightforward yet highly efficient approach that effectively leverages both long reads and co-barcoded reads for haplotype-resolved assembly.

## 1 Introduction

A complete genome assembly provides a comprehensive blueprint of an organism’s genetic material, facilitating accurate identification and characterization of genetic variants, and serving as a valuable reference for the discovery of conserved sequences and evolutionary relationships between species. Genome assembling computationally reconstructs genomes by identifying overlaps among genomic sequencing reads. Most assemblers employ assembly graphs to simplify the process of genome reconstruction, reducing it to a path problem. However, the intricate presence of repetitive genomic regions leads to entanglements within the assembly graphs and yielding fragmented and haplotype-collapsed genomes, as seen in the current human reference genome, GRCh38 (Popejoy and Fullerton, 2016; Ballouz et al., 2019). The recent achievement of the telomere-to-telomere (T2T) human reference genome by collaborative efforts such as the T2T Consortium and the Human Genome Structural Variation Consortium (HGSVC), represents a significant leap forward in genome assembly (Miga et al., 2020; Rhie et al., 2023). By leveraging cutting-edge sequencing technologies and employing paired assembly algorithms, these initiatives have successfully untangled complex regions of the genome including highly repetitive sequence regions of centromeres and telomeres. Notably, the CHM13 cell line consists of two nearly identical haploid complements to eliminate the need for haplotype separation and mitigate diploid assembly errors (Yang et al., 2023). Despite these advancements, a “perfect” genome assembly should possess satisfactory contiguity, with acceptable assembly errors, while being haplotype-resolved.

Haplotyping, also known as phasing, involves determining the specific arrangement of alleles on each chromosome, thereby distinguishing maternal and paternal haplotypes (Ebert et al., 2021; Garg et al., 2021). Resolving haplotypes not only aids in discerning allele-specific expression but also facilitates the identification of cis-regulatory elements and characterization genomic variations, which enables more precise investigations into the intricate relationship between genotype and phenotype (Rhie et al., 2023). Consequently, there exists an urgent imperative to advance genome assembly methodologies that can achieve a complete diploid human genome with a T2T-level assembly of both haploid genomes that accurately capture the diversity and complexity of biological genomes (Peters et al., 2015; Jarvis et al., 2022).

Numerous laboratory and computational approaches have been developed to tackle these challenges. Recent breakthroughs in sequencing technologies such as synthetic long read (SLR) libraries, third-generation sequencing long reads (TGS), BioNano physical maps, and Hi-C contact maps, have revolutionized genome assembly methodologies (Sedlazeck et al., 2018). Leveraging their long-range information, these technologies surpass mainstream next-generation sequencing (NGS) methods, enabling the reconstruction of complex genomic regions containing repetitive elements and facilitating the generation of high-quality, haplotype-resolved, and chromosome-scale assemblies (Logsdon et al., 2020; Garg et al., 2021; Qi et al., 2022). Notably, TGS such as Pacific Biosciences (PacBio) and Oxford Nanopore Technologies (ONT) in conjunction with the recent development of computational tools, have been hailed as the “method of the year 2022” due to their promising capabilities in sequencing and assembling the complete genomes (Long-read sequencing powers a more complete reading of genomic information, 2023). However, TGS long reads have presented challenges in terms of sequencing accuracy and throughput, stemming from underlying technological principles, which have impacted the accurate identification of heterozygous variants (Wohlers et al., 2023). Recent efforts for ONT ultra-long (Jain et al., 2018) and PacBio high-fidelity (HiFi) (Wenger et al., 2019) reads hold the promise in accurately resolving whole-genome haplotyping. Additionally, recent advancements in SLR sequencing technologies, such as MGI’s single-tube long fragment reads (stLFR) (Wang et al., 2019), 10x Genomics’ linked reads (Zheng et al., 2016), UST’s TELL-Seq (Chen et al., 2020) and Loop Genomics’ LoopSeq (Callahan et al., 2021), exhibit even longer information than TGS, and have demonstrated success in genome assembly (Kuleshov et al., 2016; Guo et al., 2021b; Mak et al., 2023), haplotyping (Wang et al., 2019), and structural variation (SV) detection (Guo et al., 2021a; Liu et al., 2021). SLR co-barcoded reads represent an augmented NGS technology that entails fragmenting chromosomes into numerous long DNA fragments and assigning the same barcode to reads originating from a single fragment. Consequently, reads with identical barcodes are presumed to derive from the same extended DNA fragment, thus suggesting that their positions on the chromosome ought to be within the length range of DNA fragments and on the corresponding haplotype. Therefore, by utilizing the barcodes as indicators of the physical proximity of reads, co-barcoded sequencing enables read binning for local assembly, and has also been employed in sequencing decontamination (Xu et al., 2023), single-cell transcriptomics (Han et al., 2022; Jovic et al., 2022), and high-resolution spatial omics (Chen et al., 2022; Guo et al., 2023). However, the limited capturing efficiency of co-barcoded reads poses a significant obstacle to the direct local assembly of these read clouds, impeding the generation of high-quality contigs as achieved with TGS reads. Moreover, trio-binning-based approaches utilize heterozygous pedigree information to classify the offspring’s sequencing data into distinct maternal and paternal groups. By combining parental-specific markers with long-range information, which compensates for high sequencing error rates in TGS reads or low capture ratios in SLR reads, trio-binning-based strategies can reconstruct the entire haplotype (Koren et al., 2018; Xu et al., 2021).

The conventional long-range approach, relying solely on error-prone TGS long reads or inefficiently captured SLR co-barcoded reads, has demonstrated limited success in generating accurate, haplotype-resolved genome assemblies for large, repeat-rich human genomes. To address this challenge, a hybrid assembly algorithm that integrates long reads with precise SLR reads has emerged as a promising solution to achieve high-quality, haplotype-resolved assemblies while minimizing computational requirements. Currently, hybrid approaches to genome assembly can be classified into four main types: (a) TGS contigs + SLR polishing, which involves initially assembling contigs based on TGS reads and then using SLR’s short reads to correct errors through consensus or local assembly (Kang et al., 2023; Darian et al., 2024); (b) TGS contigs + SLR scaffolding, where TGS contigs are assembled first and then SLR’s short reads are mapped to these contigs to link them into scaffolds (Guo et al., 2021b); (c) SLR scaffolds + TGS gap-closing, which starts by assembling SLR co-barcoded reads into scaffolds and then uses TGS long reads to fill the gaps within these scaffolds (Xu et al., 2020; Schmeing and Robinson, 2023); and (d) SLR contigs/scaffolds + TGS (re-)scaffolding, which constructs SLR scaffolds initially and extends pre-assembled scaffolds using the long-range information from TGS reads (Zimin et al., 2013; Zimin et al., 2017; Di Genova et al., 2021). Different assembly algorithms are applied separately to TGS and SLR reads, taking advantage of their respective long-range benefits. SLR co-barcoded sequencing builds and iteratively trims de Bruijn graphs (DBG) as used in many modern NGS short-read assemblers that use consecutive *k*-mers to avoid the computationally expensive all-versus-all read comparison (Weisenfeld et al., 2017). On the other hand, contemporary long-read assemblers for third-generation sequencing (TGS) have widely adopted the all-versus-all read alignment approach, utilizing overlap-layout-consensus (OLC) graphs or string graphs, and leveraging novel algorithms to expedite the process of read comparison (Koren et al., 2017; Xiao et al., 2017; Kolmogorov et al., 2019). While some hybrid assembly approaches employ resource-intensive polishing or initiate assembly with short reads, they fail to adequately address the crucial issue of assembly contiguity (Ruan and Li, 2020). Despite multiple rounds of polishing, a significant number of consensus errors persist due to the inefficient utilization of valuable short-read sequence information. Consequently, these errors impede downstream genome analyses, including gene and protein prediction (Watson and Warr, 2019). Furthermore, other scaffolding or re-scaffolding approaches typically rely on pre-assembled TGS contigs or SLR scaffolds in the initial step, thereby neglecting the propagation of assembly errors induced by previous misassemblies.

To overcome the challenges in achieving the perfect genome assembly, we demonstrate a novel hybrid assembly pipeline called AsmMix, that leverages the complementary strengths of TGS long reads and SLR co-barcoded sequencing to enhance haplotype-resolved genome assembly. Unlike most existing hybrid assemblers, AsmMix integrates assemblies from co-barcoded and TGS reads independently, thus avoiding biases introduced by sequencing platforms and bioinformatics algorithms. It involves employing assembly mixing to rectify errors found in longer TGS contigs by leveraging the accuracy of co-barcoded assemblies. This strategic approach is based on identifying and addressing discrepancies between the TGS assemblies and the co-barcoded assemblies, considering them as errors on a small scale. Consequently, these errors are rectified by substituting them with sequences derived from the co-barcoded assemblies. Moreover, the longer phasing blocks in SLR assemblies guide the reconstruction of haplotypes with or without trio information. When applied to both synthetic and real datasets, it consistently maintains high-quality assembly contiguity and haplotyping accuracy, outperforming other competing tools. Furthermore, this approach allows us to assemble the uniformly heterozygous HG002 cell line with high accuracy and provides phased information for both haploid genomes. This innovative approach extends beyond accurate and complete genome assemblies, which has the potential to revolutionize personalized medicine by enabling precise genomic analyses and deepens our understanding of genetic disorders, population genetics, and evolutionary processes.

## 2 Methods

**FIGURE 1:**
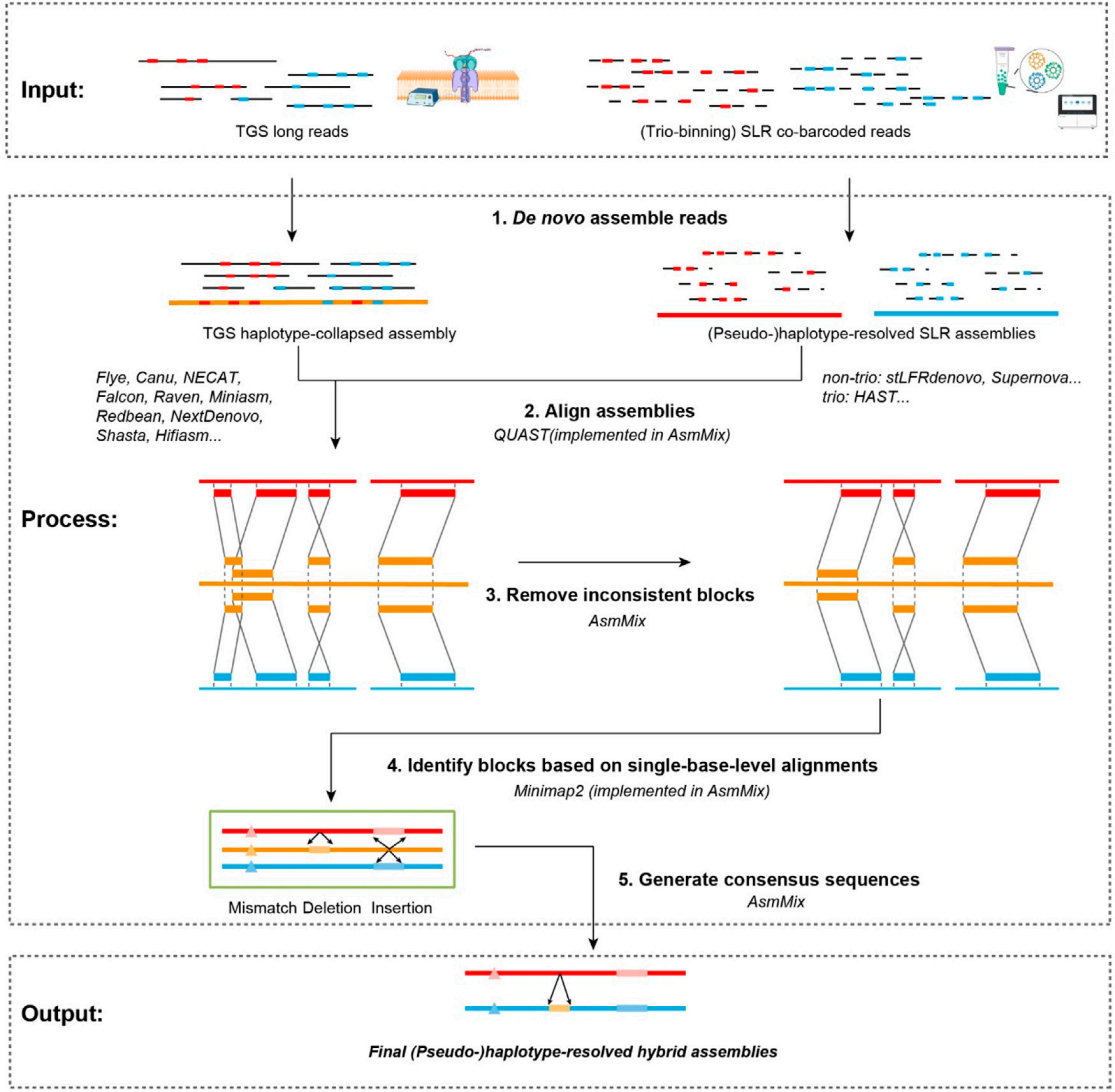
Workflow of AsmMix. AsmMix requires both TGS long reads and SLR co-barcoded reads as input, generates preliminary haplotype-collapsed assemblies using long reads and haplotypes using trio binning and barcode cloud information, merges two sets of genome sequences, and finally reconstructs the haplotype-resolved assemblies. The gray lines indicate sequencing long reads and long fragments data (solid and dashed), the red and blue blocks refer to maternal and paternal allelic sites, the red and blue lines refer to reconstructed maternal and paternal haplotypes, and yellow lines and blocks represent the haplotype-collapsed assembly and regions, respectively.

The AsmMix pipeline is designed to utilize TGS and SLR reads as input and is compatible with mainstream long-read and co-barcoded-read assemblers. The main steps are depicted in Figure 1. It initiates by executing SLR assemblers on the co-barcoded reads, thereby producing a collection of haplotype-resolved assemblies. At the same time, it concurrently performs the haplotype-collapsed TGS assembly by Flye (Kolmogorov et al., 2019), NECAT (Chen et al., 2021), or other *de novo* TGS assemblers. If the trio information is available, HAST (Xu et al., 2021) can be utilized to obtain two complete haplotypes. Alternatively, SLR assemblers like stLFRdenovo (https://github.com/BGI-biotools/stLFRdenovo) and Supernova (Weisenfeld et al., 2017) can generate two pseudohaps for subsequent mixing. The next step involves the mixing of these SLR and TGS assemblies. This step is to rectify short-range errors present in the TGS assembly by leveraging the information obtained from the SLR assembly. First, AsmMix compares one of the haplotype-resolved SLR assemblies against the TGS assembly to obtain a list of alignment blocks by individually mapping the stLFR assembly against the TGS assembly. Indeed, we use Minimap2 (Li, 2018) to align each assembly pair and run QUAST (Gurevich et al., 2013) to generate, score, and select the optimal set of alignment blocks. Subsequently, the pipeline proceeds to perform a thorough screening of the generated set to identify and eliminate potential inconsistencies. This screening involves conducting base-level pairwise alignments using Minimap2 for each alignment block. During this process, the pipeline examines the substitutions and insertion-deletion (Indels) events between the two sequences being compared. A specified threshold is employed (default: 50 bp) to determine errors in the TGS assembly. Any substitution or Indel event with a length below this threshold is categorized as an error and subsequently replaced with the corresponding sequence from the SLR assemblies. Conversely, substitutions/Indels that exceed the threshold are disregarded and not subjected to correction. This strategic approach effectively targets and corrects the majority of short-scale errors present in the TGS assembly. By replacing erroneous regions with their corresponding sequences from the SLR assemblies, the pipeline ensures enhanced accuracy and integrity of the final assembly. To retain both haplotypes, each haplotype from the SLR assembly is independently mixed with the TGS assembly. This step is essential to preserve the phased information and enable further downstream analyses that rely on haplotype-resolved genomic sequences. The AsmMix pipeline is implemented in Python and is freely available at https://github.com/BGI-tianjin-dev/AsmMix. In the subsequent sections, we provide a detailed description of two core modules, screening inconsistent alignment blocks and replacing error-prone sequences.

### 2.1 Screening inconsistent alignment blocks

**FIGURE 2:**
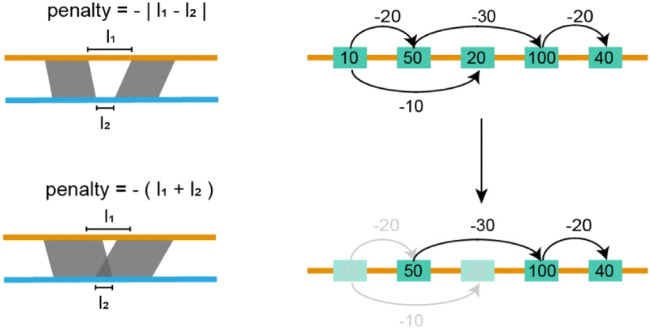
Filtering inconsistent blocks. In the figure above, each yellow rectangle indicates an alignment block, the number in each rectangle indicates the length of the alignment block, and the number on the arrow indicates the penalty score. We seek a path that maximizes the sum of lengths and penalties it passes through. In the figure below, the shallow yellow rectangles indicate discarded alignment blocks, yellow rectangles, and black lines indicated selected blocks and paths.

The screening module starts with the assembly alignment, in which the TGS assembly is set as the target while the SLR assemblies serve as the query (Figure 2). We use Minimap2 to perform this alignment, and generate a set of alignment blocks for each SLR scaffold. These blocks are then sorted based on the alignment quality on the SLR assembly and denoted by $b_{i},i=1,\ldots ,n$ . In order to assess the quality of a pair of alignment blocks $b_{i},b_{j}$ , we define a penalty function $p\left(b_{i},b_{j}\right)$ as follows: if the aligned regions of the blocks on the TGS contigs do not overlap, then the penalty is defined as $-\left|l_{1}-l_{2}\right|$ , in which $l_{1}$ and $l_{2}$ represent the gap lengths between the aligned regions on the SLR scaffolds and TGS contigs, respectively. On the other hand, if the aligned regions on the TGS contigs overlap, then the penalty is $-\left(l_{1}+l_{2}\right),$ in which $l_{1}$ is gap length between the aligned regions on the SLR scaffolds and $l_{2}$ is the length of the overlap on the TGS contigs. For a chain of alignment blocks $\left\{b_{k_{1}},{\ldots ,b}_{k_{m}}\right\},k_{1}<\ldots {<k}_{m}$ , the score is defined as:

$$s=\sum_{i=1}^{m}len\left(b_{k_{i}}\right)+\sum_{i=1}^{m-1}p\left(b_{k_{i}},b_{k_{i+1}}\right)$$

in which $len\left(b_{k_{i}}\right)$ represents the length of the aligned region on the SLR scaffolds. It is important to note that if a block is mapped to distinct TGS contigs, or if their strands are different, or if the length of their overlap on the TGS contigs exceeds a preset ratio (80% of the length of the shorter aligned region by default), then the penalty is set to −1,000,000,000. To select the chain that maximizes the score s , we apply a dynamic programming algorithm. We define $s_{i}$ as the maximal score for chains terminated at block $b_{i}$ , and then we determine $s_{i}$ using the following recursive relation:

$$s_{i}=len\left(b_{i}\right)+\max\left(0,\max_{j=1,\ldots ,i-1}\left(s_{j}+p\left(b_{i},b_{j}\right)\right)\right)$$

Thus if $s_{i}$ is the maximal score, then the optimal chain must be terminated at $b_{i}$ and the whole chain can be recovered through a trace-back process. During implementation, we conduct a two-round iteration to ensure the integrity of the analysis, considering both the positive and negative strands. This ensures that we take into account alignment blocks on both strands of the DNA sequence. Once the optimal chain of alignment blocks has been established, we utilize stringent measures to ensure their congruence with respect to the target sequences and strand orientation. Alignment blocks that are found to be mapped to disparate targets or divergent strands are flagged and passed on to the subsequent sequence replacement module for further examination and potential correction.

### 2.2 Replacing error-prone sequences

Following the screening module, the AsmMix pipeline employs a rigorous clustering approach to organize alignment blocks according to their corresponding TGS contigs. Within each TGS contig, the alignment blocks are sorted based on their starting positions to ensure that the blocks are arranged in a logical order. Subsequently, a comprehensive analysis is conducted on each TGS contig, whereby all alignment blocks undergo an examination to eliminate any instances of overlapping between consecutive regions. This critical step is executed to ensure that each region is unique and non-redundant in accordance with the following rules: (a) if one region encompasses the subsequent region, the latter is discarded as it is redundant; (b) if two regions are found to overlap, priority is assigned to the latter region, assimilating the overlapping areas. Once the overlapping regions have been resolved, the AsmMix pipeline proceeds to subject the selected regions from each TGS contig, along with their corresponding counterparts from the alignment blocks, to a pairwise alignment using the Minimap2 algorithm. During this alignment, AsmMix scrutinizes the *cs* tags within the PAF (Pairwise mApping Format) files. These tags provide detailed information about the alignment, such as insertions, deletions, and substitutions. In the final replacement stage, the AsmMix pipeline disregards any *N* bases present in the SLR assemblies. Additionally, a carefully chosen length threshold (50 bp by default) is used to determine whether a replacement should occur when an Indel signal is detected within the *cs* tag. This threshold ensures that only significant insertions or deletions are considered for replacement. Ultimately, the pipeline concatenates the replaced sequences with the sequences originating from regions not covered by alignment blocks, culminating in a comprehensive and cohesive output that combines the information from both the alignment blocks and the original TGS contigs, providing a more accurate representation of the targeted genomic regions.

### 2.3 Benchmarking datasets

To quantitatively assess the performance of the *de novo* genome assembly achieved by AsmMix, we generated simulated data based on the HG002 reference genome using PBSIM2 (Ono et al., 2021) (Supplementary Table S1). We chose HG002 rather than the single human reference genome (Shumate et al., 2020), GRCh38 because the latter is a mosaic genome derived from multiple individuals and lacks pedigree information. Instead, HG002 derived from the Ashkenazi trio (HG003 as the father and HG004 as the mother), is an NIST reference genome sourced from Genome In A Bottle (GIAB) (Zook et al., 2016). The availability of the parental genomes enables the evaluation of haplotyping effects in assemblies. The reference (GCA_021950905.1_HG002.pat.cur.20211005_genomic.fna) was downloaded online, offering greater continuity and completeness compared to GRCh38 (Jarvis et al., 2022). To expedite the benchmarking process, we specifically extracted Chromosome 19 (Chr19) and performed individual simulations of error-free TGS reads, ONT reads, PacBio CLR and HiFi reads. In addition, we also assessed the impact of read coverage, sequencing error rate, and read length on the mixed assembly. The generative model employed to assign quality scores and error profiles varied based on the sequencing platforms employed (R95 chemistry for ONT and P6C4 chemistry for PacBio; substitution: insertion: deletion = 23:31:46 for ONT and 6:50: 54 for PacBio). Note that there has been no SLR read simulator available. Hence, we directly extracted stLFR co-barcoded reads that were mapped to the Chr19 reference genome for assembling and benchmarking.

We also downloaded 24, 915, 207, 810 bp ONT PromethION long reads, 65, 228, 232, 554 bp PacBio Sequel I long reads, and 74, 559, 455, 800 stLFR co-barcoded reads for a plant genome*, Macadamia jansenii* (Murigneux et al., 2020). In this case, the publicly available reference genome of *Macadamia integrifolia* v2 (Genbank accession: GCA_900631585.1) was obtained as a reference for the QUAST benchmarking.

### 2.4 Validation methods

To assess the genome assemblies for the simulated datasets, we extracted the Chr19’s reference genome from the HG002 reference. QUAST v5.0.2 was utilized to report the assembly statistics, encompassing various metrics such as total length, contig NG50, contig NGA50, genome fraction, misassemblies, and local misassemblies. The default parameters were employed, except for the -m parameter set to 1,000. The genome’s completeness and quality was also evaluated by BUSCO v4 with the marker set *primates_odb10* (Manni et al., 2021).

In addition, Merqury (Rhie et al., 2020) was applied for benchmarking the precision and recall of haplotyping. This assessment was carried out by utilizing reliable haplotype-specific *k*-mers derived from the sequencing reads of the trio. The contiguity was measured by the maximum length and N50 length of contigs. To determine the single-base assembly quality value (QV) and completeness, haplotype-specific *k*-mers (hapmers) were generated by intersecting parental-specific k-mer sets with the offspring’s read set. The QV was calculated based on the survival rate of *k*-mers, considering only those found exclusively in each haplotype-resolved assembly as assembly errors. Completeness was determined by comparing the fraction of recovered solid *k*-mers in the assembly to the offspring’s sequencing reads. In addition, the haplotyping precision was given by

$$Haplotyping\;Precision=\frac{correctly\;found\;hapmers\;in\;an\;assembly}{totally\;found\;hapmers\;in\;an\;assembly}$$

The haplotyping recall rate was determined by

$$Haplotyping\;Recall=\frac{correctly\;found\;hapmers\;in\;an\;assembly}{total\;hapmers\;for\;the\;offspring}$$

The haplotyping F1-score was then calculated using the formula

$$F1-score=\frac{2\cdot Haplotyping\;Precision\cdot Haplotyping\;Recall}{Haplotyping\;Precision+Haplotyping\;Recall}$$

Phasing blocks, which consist of hapmers from the same haplotype, were identified by the presence of more than two hapmers in the assembly. Conversely, the switch error rate was determined by the observation of hapmers from the other haplotype within a window of the phasing blocks (20 Kbp by default).

### 2.5 Other tools

We used default parameters for TGS long-read assemblers including Flye and Canu, and SLR co-barcoded assemblers including stLFRdenovo (Supplementary Table S6). To evaluate the performance of hybrid assemblies, we also utilized WENGAN (Di Genova et al., 2021), a state-of-the-art hybrid assembler, in both its fastest WENGAN-M (MINIA3) and most contiguous WENGAN-D (DISCOVARdenovo) modes. Furthermore, we employed TrioCanu (Koren et al., 2018) and HAST to benchmark the accuracy and efficiency of haplotyping using the same dataset. It is important to note that TrioCanu and HAST employed the same trio-binning strategy to identify paternal-specific and maternal-specific reads for generating haplotype-resolved genome assemblies. However, they exclusively relied on TGS long reads or SLR co-barcoded reads.

## 3 Results

### 3.1 Hybrid assemblies of synthetic datasets

**FIGURE 3:**
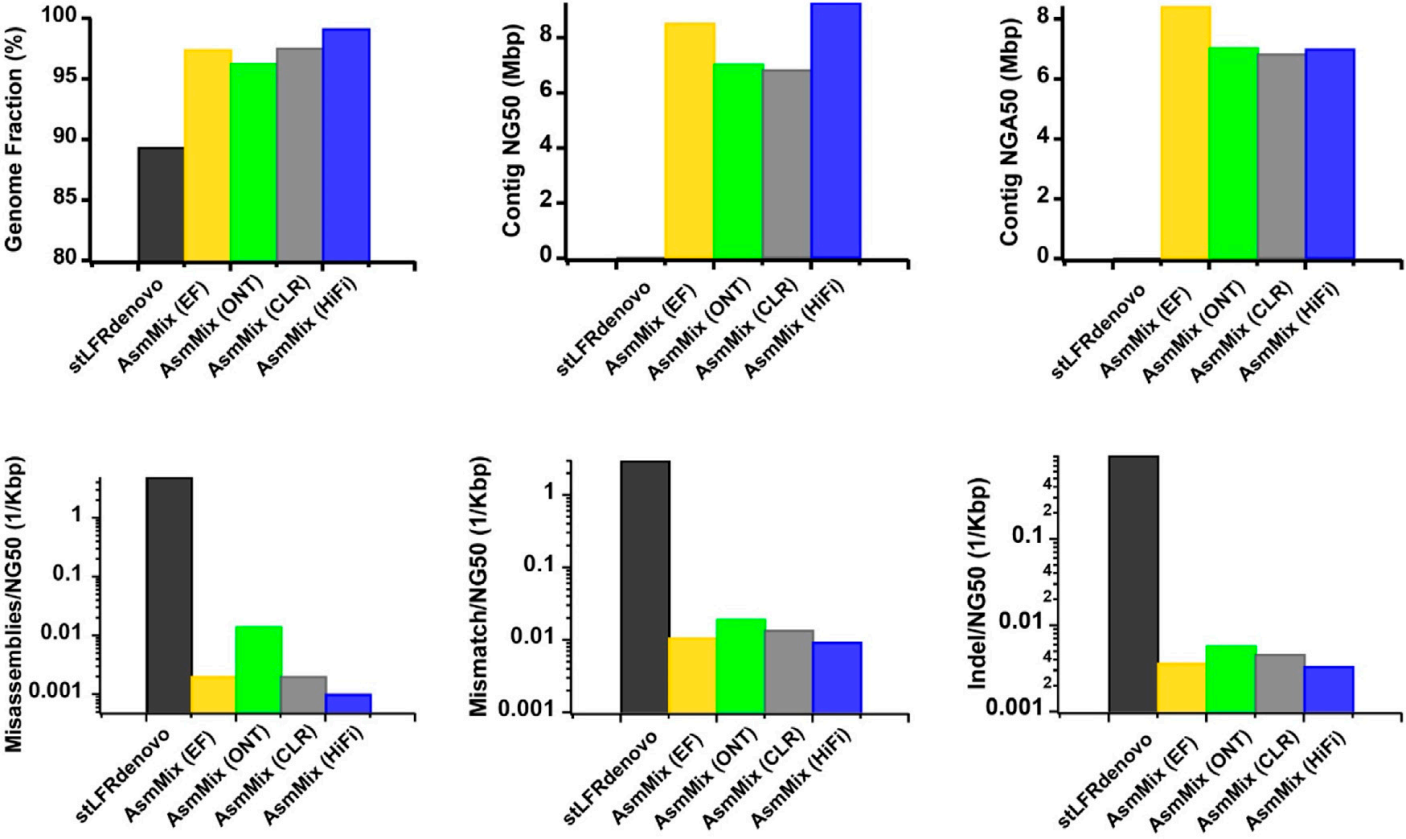
Improved assemblies using simulated data for different TGS sequencing platforms. The metrics were measured by QUAST.

AsmMix accepts all types of main TGS and SLR data types. To assess the robustness, we simulated error-free (EF), ONT, PacBio CLR, and HiFi reads, each with different read length distribution and sequencing error patterns, and individually assembled them with stLFR reads. Overall, all four hybrid assemblies achieved a minimum genome fraction of 95%, effectively covering the majority of the reference genome (Figure 3). In contrast, the stLFRdenovo assembly, which utilized the same stLFR reads exclusively, only recovered 89.340% of the reference regions. In terms of contiguity, the PacBio HiFi hybrid assembly exhibited the longest contig NG50 values, followed by the EF, ONT, and CLR assemblies. The EF hybrid assembly outperformed the others in terms of contig NGA50 values when aligned against the reference. This may be attributed to the relatively high single-base sequencing accuracy of EF and PacBio HiFi reads, resulting in simpler assembly graphs. Despite having the highest sequencing error rate, ONT outperformed PacBio CLR in terms of contig NG50 and NGA50 values, which can be attributed to its relatively longer read length advantage. This longer read length aids in resolving repetitive regions, thereby improving assembly quality. The variations in sequencing accuracy observed across different sequencing platforms were consistent with the occurrence of long-range misassembles, as well as short-range mismatches and Indels. It is worth noting that we normalized these values by their contig NG50 to mitigate the impact of assembled sequence length. Among the evaluated hybrid assemblies, EF and PacBio HiFi hybrid assemblies exhibited the fewest misassembles, mismatches, and Indels due to the higher single-base accuracy of TGS reads. These observations underscore the robustness of AsmMix, which is unaffected by TGS read length and accuracy, and consistently achieves high-quality assemblies across different sequencing platforms.

### 3.2 Comparison with other hybrid assemblers

**FIGURE 4:**
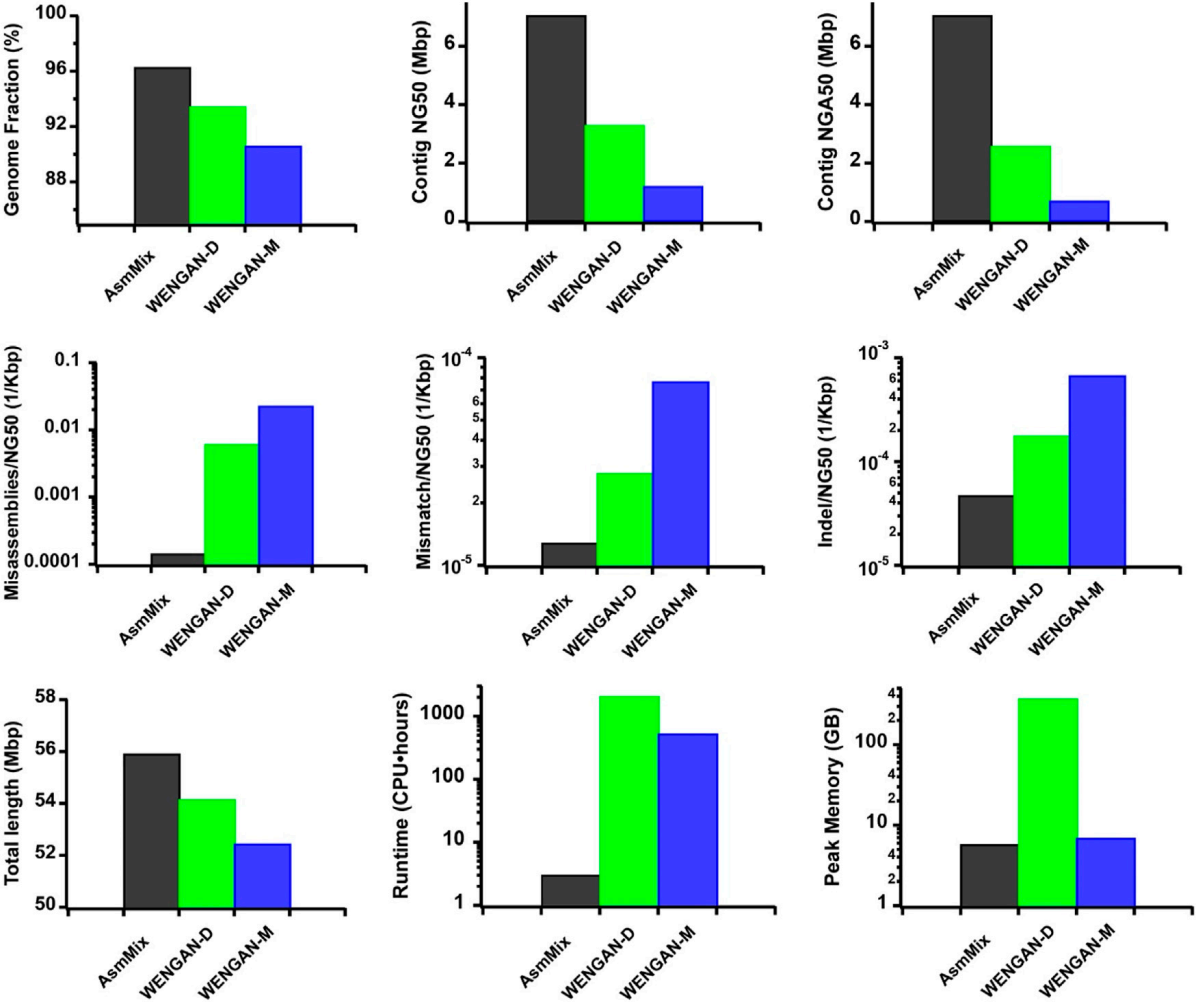
Comparison with different hybrid assembles. The metrics were measured by QUAST. We benchmarked their performance on a Linux system with Intel Core Processor (Broadwell, IBRS), 12 CPU cores and 24 threads. It is worth noting that AsmMix relies on pre-assembled long-read and co-barcoded assemblies, which entail additional computational consumptions.

In our comparison with other state-of-the-art hybrid assemblers using the same ONT and stLFR synthetic datasets, AsmMix demonstrated remarkable performance in terms of total assembly length and coverage of the HG002 Chr19 reference genome (Figure 4). Notably, AsmMix achieved a 2.13-fold and 2.75-fold increase in contig NG50 value compared to WENGAN-DiscovarDenovo (WENGAN-D) and WENGAN-Minia3 (WENGAN-M), respectively, while its NGA50 value was 2.73-fold and 3.68-fold higher, respectively. Furthermore, our hybrid algorithm significantly reduced long-range misassembles by more than two and three orders of magnitude compared to WENGAN-D and WENGAN-M, respectively. Additionally, AsmMix exhibited the fewest short-range assembly errors, including mismatches and Indels, among the three assemblers.

Moreover, a comprehensive comparison of computational resources would provide users a clear understanding of the feasibility of implementing AsmMix in their own work (Garg, 2021). Thus, we compared the computational consumptions of different hybrid assemblers. AsmMix outperformed the other assemblers in terms of computational resources, running considerably faster than WENGAN-D and WENGAN-M due to its straightforward yet highly efficient hybrid algorithm design. Its peak memory usage was similar to that of WENGAN-M, but much lower than that of WENGAN-D.

### 3.3 Comparison with other haplotype-resolved assemblers

We evaluated the performance of four state-of-the-art haplotype-resolved assemblers using the same datasets and utilized Merqury to assess the haplotyping effect. AsmMix, HAST, and TrioCanu employed a binning strategy to separate reads and generate haplotype-resolved datasets. However, it is important to note that HAST was specifically designed for SLR reads, while TrioCanu exclusively utilized TGS long reads. We also included stLFRdenovo, which does not rely on trio-binning and instead generates two pseudo-haplotypes. AsmMix surpasses the stLFR co-barcoded assembly in terms of contig N50/NG50 length (Table 1). This is because the co-barcoded assemblers face limitations due to the low coverage of read pairs from the same long fragment. Consequently, they struggle to fully reconstruct the complete long fragment using mainstream co-barcoded sequencing platforms, resulting in fragmented assemblies with many N’s left in the scaffolds. To address this issue, AsmMix integrates TGS long reads into the assembly process. It utilizes the long-read assembly as the backbone sequence and connects the haplotype-resolved sequences assembled by stLFR co-barcoded reads in the same genomic region. By doing so, the contig N50/NG50 length in the AsmMix assembly benefits from the inclusion of the TGS assembly, which is significantly longer compared to stLFRdenovo and HAST assemblies. In specific scenarios, AsmMix even outperforms the long-read assembly generated by TrioCanu, as TrioCanu struggles with the inefficiency of trio binning on long reads due to high sequencing error rates. AsmMix overcomes this limitation by utilizing accurate co-barcoded reads for trio binning, effectively replacing heterozygous regions in the long-read assembly and ensuring the generation of longer contigs. On the other hand, stLFRdenovo and HAST, which were based on SLR reads, demonstrated higher *k*-mer-based QV due to the increased sequencing accuracy of SLR reads. The completeness values of all assemblers were above 90%, except for TrioCanu, as the high sequencing error rates hindered the accurate identification of hapmers.

**TABLE 1:** Haplotype-resolved assembly quality statistics.

Assembly	Contiguity		Quality		Haplotyping		
	Contig max (bp)	Contig N50 (bp)	QV (Phred)	Completeness (%)	Precision (%)	Recall (%)	F1-score (%)
**AsmMix**							
Paternal	2,762,169	938,888	34.09	93.18	72.65	49.14	58.63
Maternal	2,491,016	614,713	34.88	93.47	73.20	48.47	58.32
Combined	2,762,169	739,405	34.47	97.89	94.27	51.79	66.85
**stLFRdenovo**							
Pseudohap1	376,611	51,558	57.83	93.77	53.36	36.76	43.53
Pseudohap2	376,608	51,543	57.99	93.79	46.79	37.04	41.35
Combined	376,611	51,547	57.91	95.25	74.06	49.81	59.56
**HAST**							
Paternal	349,597	36,239	54.16	92.99	78.17	46.52	58.33
Maternal	278,083	33,038	53.54	92.18	72.94	46.05	56.46
Combined	349,597	34,464	53.90	97.06	98.26	47.11	63.69
**TrioCanu**							
Paternal	498,940	122,721	26.71	62.48	64.99	34.89	45.41
Maternal	1,157,749	227,689	27.96	77.19	51.79	37.75	43.67
Combined	1,157,749	162,254	27.35	95.09	82.35	44.01	57.36

Haplotyping errors arise from the presence of unexpected hapmers in the assembly. The haplotype precision of AsmMix for paternal-specific and maternal-specific assemblies was 72.65% and 73.20%, respectively. In contrast, stLFRdenovo pseudo-haplotypes exhibited haplotype precisions of only 53.36% and 46.79%, while TrioCanu haplotypes had haplotype precisions of 64.99% and 51.79%. HAST accurately detected expected hapmers, indicating that the inclusion of long-range information from SLR reads contributes to improved global haplotyping precision. AsmMix demonstrates comparable precision and recall in haplotyping when compared to HAST results. This similarity can be attributed to the fact that both tools employ trio-binning stLFR data for haplotyping. By comparing the number of expected hapmers present in the child’s diploid assembly, we observed that each haplotype generated by different assemblers recovered the same level of parental heterozygous sites. The major strength of AsmMix lies in its ability to maintain the excellent contiguity of TGS assembly while incorporating the robust haplotyping performance of co-barcoded reads.

**FIGURE 5:**
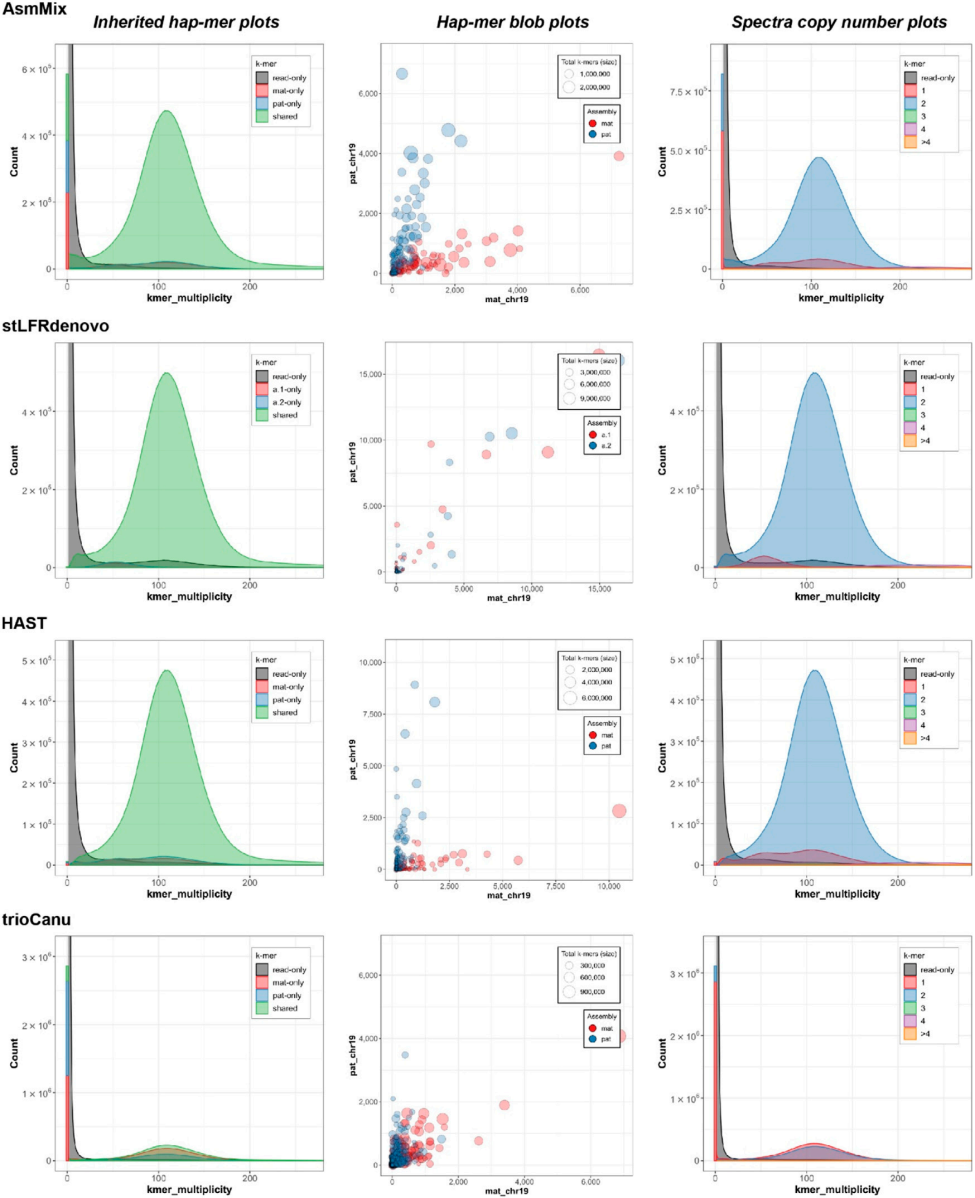
Evaluation of haplotyping with characteristic *k*-mers. The inherited hapmer plots (left) measured the hapmer distribution and overlying with the offspring’s reads. The hapmer blob plots (middle) were used to assess the overall phasing across each assembly. Each dot (circle) represented a sequence (contig or scaffold) with its size relative to the sequence length. The *x* and *y*-axis were the number of detected hapmers. The spectra copy-number plots (right) provided the copy-number analysis. These figures were rendered by Merqury.

In the stacked haplotyping assembly spectra of *k*-mer multiplicity, the main peak (green) represents *k*-mers that are shared by both haplotypes, and its height corresponds to the sequencing depth (Figure 5). The smaller blue and red peaks indicate *k*-mers that occur in only one of the haplotype-resolved assemblies, and their x-values should be half of the main peak or even integral multiples. AsmMix, stLFRdenovo, and HAST haplotype-resolved assemblies exhibited the expected patterns in the spectra. However, the high sequencing error rate of TGS long reads affected the genome reconstruction using TrioCanu, resulting in overlapping peaks.

By incorporating long-range information from SLR co-barcoded read data, the pipeline experienced improved contiguity and precision in the phase blocks of the assemblies. One of the challenges in haplotype assembly is the correct grouping of haplotype-specific variants. Failure to accurately group these variants can result in switch errors, where haplotypes are mistakenly swapped within a contig. Switch errors can lead to the splitting of contigs and a reduction in the size of haplotype blocks. AsmMix achieved phase block lengths with N50 values of up to 234 Kbp and 310 Kbp for paternal and maternal haplotypes, respectively, with the longest block reaching 2.42 Mbp (Table 2). SLR-based stLFRdenovo and HAST demonstrated lower switch error rates, while TGS-based TrioCanu had a switch error rate of almost 20%. AsmMix effectively mitigated these error rates and reduced them to 15% by leveraging the advantages of both data types. The observations regarding contiguity and precision in the phase blocks of the assemblies were supported by the hapmer blob plots (Figure 5). In AsmMix and HAST assemblies, the hapmer blob plots showed near-perfect separation of hapmers across all contig or scaffold lengths. This indicates that the haplotype-specific *k*-mers were accurately assigned to their respective haplotypes, resulting in precise and contiguous phase blocks. The clear separation of hapmers in the plots suggests that the assembled haplotypes were distinct and accurately captured. Conversely, numerous contigs or scaffolds in each pseudo-haplotype assembled by stLFRdenovo and haplotypes assembled by TrioCanu shared both paternal and maternal-specific *k*-mers.

**TABLE 2:** Statistics for phase blocks and switch error rates.

Phasing blocks	No. of blocks	Genome covered (bp)	Block size max	Block size N50	Switch (%)
**AsmMix**					
Paternal	438	42,930,019	1,375,534	233,984	17.27
Maternal	357	46,369,334	2,415,885	309,740	12.80
**stLFRdenovo**					
Pseudohap1	616	46,953,031	1,430,655	340,227	6.34
Pseudohap2	594	46,112,517	1,296,386	312,444	6.98
**HAST**					
Paternal	777	43,063,437	1,411,352	310,566	7.27
Maternal	894	39,913,896	1,880,279	495,639	8.57
**TrioCanu**					
Paternal	585	21,752,871	276,945	79,735	19.45
Maternal	561	30,967,055	535,430	121,156	22.74

In the spectra copy-number plots (Figure 5), different colors represent different copy numbers of *k*-mers in the genome. The small red peak represents the 1-copy *k*-mers. These *k*-mers are specific to either the paternal or maternal genome and do not have any duplicates within a haplotype. The presence of 1-copy *k*-mers indicates sequences that are exclusive to a specific haplotype, allowing for unambiguous haplotype assignment. On the other hand, the larger blue peak corresponds to the 2-copy *k*-mers. These are sequences that are shared between both haplotypes or may exist as duplications within a haplotype. The higher copy number peaks (green, purple, orange) represent repetitive regions in the genome. These regions have multiple copies of the same sequence, which can contribute to the higher peak heights. The gray *k*-mers indicate sequences that are present in the sequencing reads but could not be assembled into the genome. These may be due to sequencing errors or missing genomic regions that were not captured in the assembly. The blue and red *k*-mers in the spectra indicate assembly errors in the paternal and maternal haplotypes, respectively. These *k*-mers represent sequences that were incorrectly assembled or merged with other sequences during the assembly process. The relatively small bar at the zero multiplicity in the *k*-mer analysis for SLR-based stLFRdenovo and HAST, assemblies indicates a high single-base-level QV., this means that these assemblies have a low error rate at the individual base level, resulting in a smaller number of *k*-mers that are found in the assembly but absent from the sequencing reads. However, the usage of error-prone long reads, such as in TGS, sequencing, can lead to higher error rates, resulting in the higher blue and red peaks at the zero multiplicity. These peaks represent *k*-mers that were erroneously duplicated or deleted during the assembly process due to sequencing errors.

### 3.4 Effect of TGS sequencing coverage, read length, and accuracy

**FIGURE 6:**
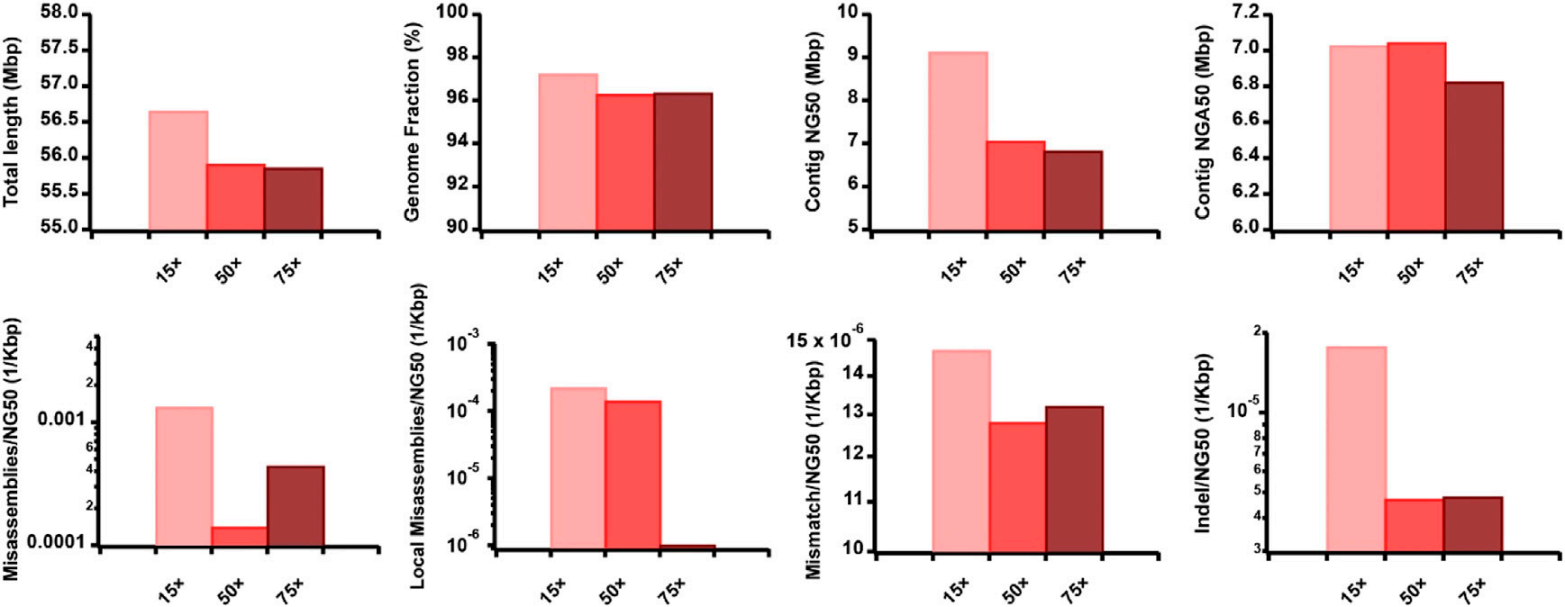
Effect of TGS sequencing coverage on the hybrid assembly. The metrics were measured by QUAST.

To evaluate the effect of TGS sequencing coverage, read length, and accuracy on the AsmMix hybrid assembly, we simulated the ONT datasets but varied their coverage depth, read length distribution, and sequencing error patterns (Supplementary Tables S2, S3). Specifically, we examined the performance of three datasets with long-read features at 15 × , 50 × , and 75 × coverage depths in reconstructing the Chr19 genome, achieving genome fraction ratios exceeding 96% (Figure 6). It is counterintuitive that the 15 × dataset generated relatively longer contig NG50 and NGA50 values compared to the higher coverage depths. This unexpected outcome suggests that deeper sequencing may introduce increased complexity into the assembly graph. However, when assessing assembly accuracy, the 75 × dataset exhibited the lowest numbers of long-range misassembles, short-range local misassembles, single-base mismatches, and Indels among the three datasets. This improvement can be attributed to the substantial enhancement in single-base accuracy resulting from the overlapping of higher sequencing data.

**FIGURE 7:**
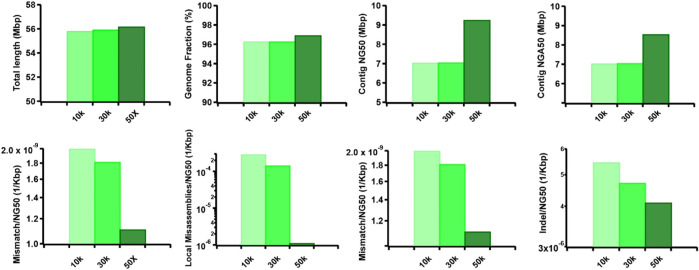
Effect of TGS read length on the hybrid assembly. The metrics were measured by QUAST.

**FIGURE 8:**
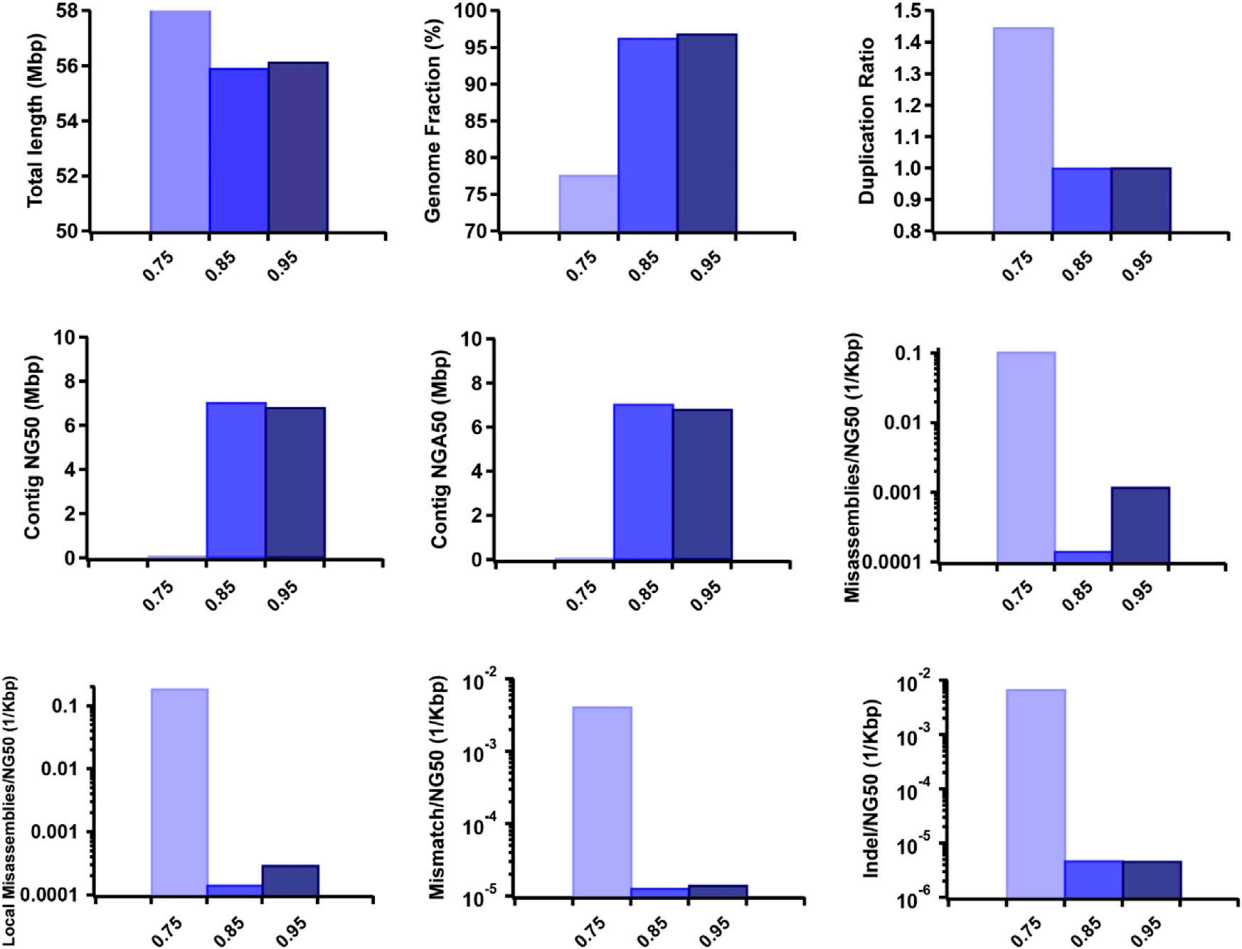
Effect of TGS sequencing accuracy on the hybrid assembly. The metrics were measured by QUAST.

Furthermore, we observed that the dataset featuring longer read lengths (mean length of 50 Kbp) outperformed assemblies with shorter read lengths (10 Kbp and 30 Kbp) across all evaluation metrics (Figure 7). This finding underscores a strong positive association between read length and assembly completeness, contiguity, and accuracy. Notably, the quality of sequencing accuracy emerged as a critical factor impacting assembly performance. The genome fraction, duplication ratio, contig NG50, contig NGA50, misassembles, local misassembles, mismatches, and Indels all demonstrated dependency on sequencing accuracy. It was evident that higher sequencing error rates induced false overlapping relationships between long reads, ultimately leading to incomplete, fragmented, and erroneous assembled sequences (Figure 8). In addition, we calculated the computational usage for different hybrid assemblers using these datasets (Supplementary Table S4). The results displayed that AsmMix still required the least CPU time among three assemblers across datasets with different parameters. In summary, our experiments demonstrate the robustness of our algorithm in accommodating diverse sequencing coverage, read length, and accuracy of long reads. Moreover, we provide valuable insights into the minimum requirements for achieving efficient hybrid assembly. These findings underscore the importance of sequencing parameters.

### 3.5 Application to a human whole genome

We applied the AsmMix pipeline to a dataset comprising 84 × stLFR SLR reads and 50 × ONT reads of HG002 sourced from the GIAB project (Zook et al., 2016), aiming to showcase the successful application of hybrid assembly for a human whole genome. The ONT reads were pre-assembled by NECAT and subsequently mixed with stLFRdenovo-assembled stLFR sequences using AsmMix. To assess the quality of the assemblies, we conducted evaluations using QUAST and the primate gene marker set *primates*_*odb10* by BUSCO v4. The results were listed in Table 3. It is evident that most metrics did not exhibit substantial changes after mixing, indicating the resilience of the AsmMix pipeline. However, it is noteworthy that the number of Indels decreased from 0.39 to 0.31 per 1 Kbp post-mixing, suggesting a reduction in Indel errors resulting from the mixing process.

**TABLE 3:** Quality assessment of *de novo* assemblies of HG002. StLFRdenovo hap1/2 is the haplotype-resolved assembly built from the stLFR dataset, NECAT is the assembly built by long reads, and AsmMix hap1/2 is the haplotype-resolved assembly after assembly mixing.

	stLFRdenovo hap1	stLFRdenovo hap1	NECAT	AsmMix hap1	AsmMix hap2
**Total length (bp)**	2,899,262,788	2,899,974,893	2,880,654,213	2,880,459,545	2,880,632,458
**Scaffold NG50 (bp)**	21,283,539	21,282,592	-	-	-
**Contig NG50 (bp)**	85,008	84,928	34,759,314	34,757,611	34,759,622
**Genome fraction (%)**	90.688	90.636	97.815	97.814	97.810
**Duplication ratio**	1.058	1.059	1.009	1.008	1.009
**Scaffold NGA50 (bp)**	1,826,678	1,826,238	-	-	-
**Contig NGA50 (bp)**	82,847	83,028	15,981,976	15,594,006	15,594,501
**# Misassemblies**	4,519	4,493	2,536	2,548	2,539
**Mismatch per 1Kbp**	1.0174	1.0128	1.2765	1.2888	1.2867
**Indel per 1Kbp**	0.2504	0.2494	0.3932	0.3116	0.3114
**BUSCO completeness (%)**	86.3	86.1	89.4	89.9	89.9

To further elucidate the accuracy of assembly, we performed variant calling and compared the results against a well-established benchmark: the GIAB benchmark encompassing small variant (single nucleotide polymorphism (SNP) and Indel) v3.3.2 and SV v0.6. The accuracy of the variant calling reflected the assembly accuracy. The variant calling from assemblies was performed by Minimap2 and PAFtools (Li, 2021). For haplotype-resolved assemblies, variations were called separately and combined using VCFtools (Danecek et al., 2011). Evaluations against benchmarks were performed by Rtg-tools (Cleary et al., 2015) for small variants and Truvari (English et al., 2022) for large SVs. Regarding SVs, the comparison between calls and benchmarks was conducted using Truvari with the parameter “-r 1,000 -passonly” and false-positive Indels were counted by a customized Perl script, in which variants with reference sequence longer than alternative sequence were considered as deletions and otherwise insertions. Variants with “N” in the alternative sequence were excluded.

We conducted a thorough evaluation of haplotyping accuracy in terms of the short/long switch error rate and phasing block N50. To assess the accuracy, we selected common heterogeneous SNPs and compared their phases. The phased SNP ratio, which represents the ratio of common heterogeneous SNPs against heterogeneous SNPs in the benchmark, was used as a measure of accuracy. We defined the short switch error as the error of flipping the phase of a single variant, while the long switch error referred to the error of flipping the phase of all variants after a particular variant. To minimize the long switch error, we employed a dynamic programming scheme with a penalty of 5 times that of a switch error. The counting of short/long switch errors was performed by minimizing the penalty function $5\cdot n_{long}+n_{short}$ , where $n_{short}$ represents the number of short switch errors and $n_{long}$ represents the number of long switch errors. Phasing blocks were defined as regions delineated by long switch errors, and the phasing block N50 was calculated as the N50 of their lengths. The comprehensive results of these evaluations can be found in Table 4. Regarding the evaluation of SNPs, the mixing procedure resulted in a 3.8/3.8-fold decrease in false negatives and a 3.4/1.4-fold decrease in false positives, with/without considering haplotyping differences. In terms of Indels, mixing led to a 3.0/3.4-fold decrease in false negatives and a 3.6/4.8-fold decrease in false positives, again with/without considering haplotyping differences. When evaluating large SVs, the false negative rate for insertions/deletions remained relatively unchanged, while the false positive rate exhibited a 1.8/1.6-fold increase. Our analysis demonstrated that the mixing procedure significantly improved the accuracy of short variants, effectively correcting a large proportion of small-scale errors. However, we also observed a decline in the performance of long variant calling, which can be attributed in part to the limited resolving power of short reads in repetitive regions. Despite this, the overall metrics for haplotyping displayed a slight improvement compared to stLFR assemblies.

**TABLE 4:** Evaluation of assembly by variant calling. The values before the slash are generated by default parameters while values after are with “-squash-ploidy”, which allows matches that ignore the haplotyping difference.

Variation type	Variation subtype	Metric	stLFRdenovo	NECAT	AsmMix
**SNV**	**SNP**	TP	2,330,486/2,523,914	1,161,996/2,067,723	2,536,637/2,779,428
		FN	698,572/505,144	1,867,062/961,337	492,421/249,635
		FP	253,797/60,369	975,393/69,669	291,137/48,351
		Precision	0.9018/0.9766	0.5437/0.9674	0.8970/0.9829
		Sensitivity	0.7694/0.8332	0.4498/0.6826	0.8374/0.9176
	**Indel**	TP	334,312/377,364	162,087/284,873	358,833/408,144
		FN	125,053/82,001	297,278/174,492	100,541/51,200
		FP	72,872/29,827	355,587/232,840	97,589/48,279
		Precision	0.8210/0.9268	0.3131/0.5502	0.7862/0.9842
		Sensitivity	0.7278/0.8215	0.3529/0.6201	0.7811/0.8885
**SV**	**Insertion**	TP	1811	3,752	3,906
		FN	3,631	1779	1,659
		FP	539	343	627
		Precision	0.7707	0.9162	0.8616
		Sensitivity	0.3329	0.6784	0.7018
	**Deletion**	TP	2,750	2,420	2,539
		FN	1,449	1,690	1,537
		FP	5,983	763	1,223
		Precision	0.3148	0.7603	0.6749
		Sensitivity	0.6548	0.5888	0.6229
**Haplotyping**		Phased SNP Ratio	0.6881	-	0.7224
		Short Switch Ratio	0.0515	-	0.0502
		Long Switch Ratio	0.0008	-	0.0007
		Phasing Block N50	4,802,248	-	5,015,779

In terms of computational efficiency, AsmMix required 7,313 thread • hours with a peak memory of 352 GB when run on a high-performance computing node with 32 threads (Intel Core Processor - Broadwell, IBRS). However, it is worth mentioning that the majority of the time and memory were spent on the pre-assembly steps of NECAT and stLFRdenovo.

### 3.6 Application to a plant genome

We also extended the application of AsmMix to a plant genome to demonstrate its scalability and potential for improving assembly quality. We downloaded 32 × ONT PromethION long reads, 84 × PacBio Sequel I long reads, and 96 × stLFR co-barcoded reads for the macadamia genome with an estimated genome size of 780 Mb (Murigneux et al., 2020). The two long-read datasets were individually pre-assembled using Canu, while the stLFR dataset was pre-assembled using Supernova, as described in the original paper. AsmMix was then employed to integrate the stLFR pseudohaplotype assemblies with the haplotype-collapsed assemblies generated from the ONT and PacBio data, respectively. Due to the unavailability of a trio-binning sequencing dataset and parental reference genomes, we were unable to perform an accurate haplotyping assessment in this case.

As a result, we benchmarked the pre-assembled long-read and co-barcoded genomes, as well as the AsmMix results, using QUAST and a publicly available reference genome of *M. integrifolia* v2 (Genbank accession: GCA_900631585.1) (Nock et al., 2020). The results were listed in Supplementary Table S5, which indicated the assembly enhancement achieved by AsmMix. Specifically, the integration approach successfully combined the long contiguity provided by the long reads with the high single-base accuracy of the co-barcoded data, resulting in improved NGA50 values. More importantly, AsmMix enabled the reconstruction of two pseudohaplotypes using the long-range haplotyping information from the stLFR reads. In contrast, mainstream long-read assembles such as Canu could only generate a haplotype-collapsed assembly without additional trio-binning information. Compared to the AsmMix and Canu assemblies using ONT long reads, we observed that assemblies utilizing a higher sequencing depth of PacBio data exhibited superior local assembly accuracy, with lower numbers of local misassemblies, mismatches, and indels. The longer total genome length observed in these assemblies may be due to the presence of duplicated allelic contigs. Note that the reference genome was obtained from a closely related species, *Macadamia integrifolia*, instead of *Macadamia jansenii*. It might have contributed to the lower genome fraction and higher misassemblies observed in all the assemblies, including the AsmMix assemblies. Despite this limitation, the AsmMix approach still demonstrated improvements in assembly quality, particularly in terms of haplotyping and contiguity, as compared to the individual long-read and co-barcoded assemblies.

## 4 Discussion

Leveraging a combination of data from diverse technologies has become a prevalent approach in genome assembly. However, the question of determining the optimal strategy for effectively combining such data remains an open challenge. In this paper, we put forth a pioneering assembly pipeline that serves as a solution to this predicament, enabling the achievement of three pivotal goals: contiguity, single-base accuracy, and haplotyping, making full use of TGS and SLR reads. Importantly, our pipeline excels in delivering assembly accuracy that is sufficiently robust for enabling accurate SNP, Indel, and SV calling, thereby exhibiting competitive performance. In addition to its inherent modularity, the AsmMix pipeline offers compatibility with various assemblers for TGS and co-barcoded reads, providing users with the flexibility to customize and test their own pipelines. This feature enhances the versatility of the pipeline and empowers researchers to tailor the assembly process to their specific requirements.

Moving forward, the development of the AsmMix pipeline will focus on the following directions. Firstly, we will leverage co-barcoded reads to generate longer contigs in SLR assemblies and rectify short-range errors. However, it is important to note that this information can also be utilized to further scaffold TGS assemblies. Many existing scaffolding methods rely on read mapping to TGS assemblies, which often demands significant computational resources. To address this challenge, we will devise a novel scaffolding approach that leverages co-barcoded assemblies. By doing so, we aim to streamline the scaffolding process and reduce the computational burden associated with mapping reads to TGS assemblies. Secondly, our current implementation utilizes only co-barcoded reads for phasing. In future investigations, we will explore the integration of TGS reads to enhance the performance of haplotyping. By incorporating TGS reads into the phasing process, we anticipate improvements in accuracy and efficiency, ultimately leading to more robust haplotyping results. Third, integrating additional types of sequencing data, such as Hi-C data, could significantly improve assembly accuracy and contiguity. Hi-C data provides information about the spatial organization of the genome, allowing for the analysis of long-range haplotyping and scaffolding at the chromosome level (Garg et al., 2021). Previous algorithms such as hifiasm and DipAsm have showed that combining Hi-C and long reads can provide chromosome-scale, haplotype-resolved assembly of genomes (Cheng et al., 2021; Garg et al., 2021). By incorporating Hi-C data into the pipeline, AsmMix could leverage the long-range information and accurate short-read sequencing data to improve the scaffolding and arrangement of contigs, leading to more accurate and contiguous genome assemblies. Fourth, another promising direction for future enhancements of AsmMix is in the field of pangenomics. By integrating chromosome-scale, haplotype-resolved genomes into high-resolution pangenomes, AsmMix could allow for capturing the full genetic diversity including complex structural variations and long-range interactions within populations (Garg et al., 2022). Fifth, future enhancements could focus on optimizing the computational resources required for analyzing large and complex genomes and handling high error rates in long-read sequencing data. An improved parallel computing strategy and a more efficient long-read aligner could deliver robust and reliable assembly results. These planned advancements highlight our commitment to refining and expanding the capabilities of the AsmMix pipeline. Through our ongoing research and development, we aim to empower researchers with a comprehensive and adaptable tool for genomic assembly, scaffolding, and haplotyping analysis.

## Data Availability

The simulated PacBio HiFi, PacBio CLR and ONT datasets were based on the HG002 reference genome (https://ftp.ncbi.nlm.nih.gov/genomes/all/GCA/021/950/905/GCA_021950905.1_HG002.pat.cur.20211005/GCA_021950905.1_HG002.pat.cur.20211005_genomic.fna.gz). The real stLFR and ONT reads of HG002 have been deposited into the CNSA with the accession number CNP0000066. We also downloaded real ONT PromethION long reads, PacBio Sequel I long reads, and stLFR co-barcoded reads for a plant genome, *Macadamia jansenii*, which have been deposited in the SRA under BioProject PRJNA609013 and BioSample SAMN14217788.
