# Supplementary material for: AsmMix: an efficient haplotype-resolved hybrid de novo genome assembling pipeline
Source: Front Genet. 2024 Jul 26;15:1421565. doi: 10.3389/fgene.2024.1421565 (PMC11310137; doi:10.3389/fgene.2024.1421565)
Supplement: Supplementary file 1 [file DataSheet1.docx]

Supplementary Material

Supplementary Table 1 Dataset source

| **Organisms** | **Datatype** | **Total reads** | **Sequencing depth** | **Read length N50 (bp)** | **Avg. fragment length (kb)** | **Source** |
| --- | --- | --- | --- | --- | --- | --- |
| Human  Chr19 | Error-free | 291,536 | 50X | 13,310 | - | PBSIM2 |
| Human Chr19 | ONT | 98,636 | 50X | 31,118 | - | PBSIM2 |
| Human Chr19 | PacBio CLR | 291,473 | 50X | 13,310 | - | PBSIM2 |
| Human Chr19 | PacBio HiFi | 295,900 | 50X | 10,000 | - | PBSIM2 |
| Human Chr19 | stLFR | - | 71X | PE100 | 118.8 | Extracted from HG002 |
| Human HG002 | ONT ultra-long | 3,734,124 | 50X | 41,815 | - | https://ftp.ncbi.nlm.nih.gov/ReferenceSamples/giab/data/AshkenazimTrio/HG002_NA24385_son/Ultralong_OxfordNanopore/guppy-V3.4.5/ |
| Human HG002 | stLFR | - | 84X | PE100 | 68.8 | https://db.cngb.org/search/run/CNR0026818/ |
| Human HG003 | NGS | - | 30X | PE100 | - | https://ftp.ncbi.nlm.nih.gov/ReferenceSamples/giab/data/AshkenazimTrio/HG003_NA24149_father/BGISEQ500/ |
| Human HG004 | NGS | - | 30X | PE100 | - | https://ftp.ncbi.nlm.nih.gov/ReferenceSamples/giab/data/AshkenazimTrio/HG004_NA24143_mother/BGISEQ500/ |

**Supplementary Table 2** Benchmarking of long-read coverage effect on hybrid assemblies

|  | **15×** | | | **50×** | | | | **75×** | | |
| --- | --- | --- | --- | --- | --- | --- | --- | --- | --- | --- |
|  | **AsmMix** | **WENGAN-D** | **WENGAN-M** | **AsmMix (ONT)** | **AsmMix (PacBio HiFi)** | **WENGAN-D** | **WENGAN-M** | **AsmMix** | **WENGAN-D** | **WENGAN-M** |
| # contigs | 28 | 160 | 248 | 25 | 36 | 43 | 84 | 19 | 39 | 40 |
| Largest contig | 11,411,160 | 2,485,509 | 3,854,999 | 21,678,202 | 11,959,218 | 10,984,170 | 5,134,225 | 15,732,865 | 10,984,965 | 6,858,979 |
| Total length | 56,648,393 | 53,148,835 | 51,427,871 | 55,908,281 | 57,474,329 | 54,142,023 | 52,438,424 | 55,856,541 | 54,195,439 | 53,359,556 |
| Reference length | 59,179,939 | 59,179,939 | 59,179,939 | 59,179,939 | 59,179,939 | 59,179,939 | 59,179,939 | 59,179,939 | 59,179,939 | 59,179,939 |
| Reference GC (%) | 48.17 | 48.17 | 48.17 | 48.17 | 48.17 | 48.17 | 48.17 | 48.17 | 48.17 | 48.17 |
| N50 | 9,117,819 | 656,244 | 463,918 | 7,043,502 | 9,262,967 | 3,291,625 | 1,446,976 | 6,822,955 | 4,619,361 | 3,481,274 |
| NG50 | 9,117,819 | 577,494 | 365,374 | 7,043,502 | 9,262,967 | 3,291,625 | 1,197,708 | 6,822,955 | 3,397,671 | 2,413,076 |
| # misassemblies | 12 | 23 | 29 | 1 | 12 | 20 | 27 | 3 | 23 | 29 |
| # local misassemblies | 2 | 79 | 46 | 1 | 2 | 72 | 32 | 0 | 73 | 42 |
| Unaligned length | 160,261 | 76,807 | 19,180 | 34,437 | 0 | 31,937 | 19,275 | 34,470 | 30,630 | 27,415 |
| Genome fraction (%) | 97.213 | 91.630 | 88.779 | 96.264 | 99.128 | 93.440 | 90.580 | 96.338 | 93.561 | 92.144 |
| Duplication ratio | 1.003 | 1.000 | 1.000 | 1.000 | 1.001 | 1.000 | 1.000 | 1.000 | 1.000 | 0.999 |
| # mismatches per 100 kbp | 134.00 | 154.42 | 146.78 | 89.87 | 85.52 | 90.19 | 91.84 | 90.12 | 92.84 | 88.91 |
| # indels per 100 kbp | 161.39 | 220.08 | 206.84 | 33.18 | 30.82 | 58.11 | 80.49 | 32.87 | 57.65 | 61.92 |
| Largest alignment | 11,411,111 | 2,359,409 | 3,390,872 | 15,700,238 | 11,959,218 | 4,514,380 | 3,385,557 | 15,678,872 | 4,514,481 | 4,784,931 |
| Total aligned length | 56,419,466 | 53,051,363 | 51,387,456 | 55,736,054 | 57,433,277 | 54,072,009 | 52,411,985 | 55,768,050 | 54,132,419 | 53,284,640 |
| NA50 | 8,502,416 | 556,989 | 415,142 | 7,043,502 | 7,001,123 | 2,626,059 | 776,993 | 6,822,952 | 2,348,274 | 1,559,403 |
| NGA50 | 7,026,439 | 491,189 | 346,225 | 7,043,502 | 7,001,123 | 2,572,940 | 698,599 | 6,822,952 | 1,963,319 | 1,343,067 |

**Supplementary Table 3** Benchmarking of long-read length and accuracy effects on hybrid assemblies

|  | **Long-read length** | | | | | | **Long-read accuracy** | | | | | |
| --- | --- | --- | --- | --- | --- | --- | --- | --- | --- | --- | --- | --- |
|  | **10 Kb** | | | **50Kb** | | | **0.75** | | | **0.95** | | |
|  | **AsmMix** | **WENGAN-D** | **WENGAN-M** | **AsmMix** | **WENGAN-D** | **WENGAN-M** | **AsmMix** | **WENGAN-D** | **WENGAN-M** | **AsmMix** | **WENGAN-D** | **WENGAN-M** |
| # contigs | 44 | 81 | 97 | 17 | 31 | 46 | 945 | 2,023 | 1,908 | 34 | 39 | 112 |
| Largest contig | 13,624,614 | 5,735,175 | 5,719,664 | 15,722,595 | 10,984,232 | 7,383,449 | 362,596 | 233,152 | 113,819 | 15,697,329 | 9,538,454 | 2,951,549 |
| Total length | 55,801,976 | 53,473,783 | 52,358,271 | 56,161,993 | 54,489,377 | 53,119,506 | 68,300,848 | 36,620,731 | 25,257,122 | 56,139,857 | 54,109,040 | 51,040,808 |
| Reference length | 59,179,939 | 59,179,939 | 59,179,939 | 59,179,939 | 59,179,939 | 59,179,939 | 59,179,939 | 59,179,939 | 59,179,939 | 59,179,939 | 59,179,939 | 59,179,939 |
| Reference GC (%) | 48.17 | 48.17 | 48.17 | 48.17 | 48.17 | 48.17 | 48.17 | 48.17 | 48.17 | 48.17 | 48.17 | 48.17 |
| N50 | 7,025,055 | 1,526,541 | 1,118,756 | 9,244,484 | 5,025,827 | 2,767,958 | 81,170 | 26,060 | 16,085 | 6,805,471 | 4,838,351 | 800,601 |
| NG50 | 7,025,055 | 1,455,448 | 986,912 | 9,244,484 | 4,719,232 | 2,747,662 | 86,768 | 11,156 | - | 6,805,471 | 4,621,389 | 745,512 |
| # misassemblies | 5 | 21 | 21 | 7 | 20 | 23 | 9 | 6 | 10 | 8 | 9 | 59 |
| # local misassemblies | 2 | 73 | 40 | 0 | 72 | 37 | 16 | 66 | 27 | 2 | 16 | 153 |
| Unaligned length | 11,139 | 31,559 | 18,786 | 32,723 | 68,903 | 63,139 | 3,302,613 | 31,119 | 44,991 | 2,510 | 31,597 | 31,871 |
| Genome fraction (%) | 96.264 | 92.303 | 90.436 | 96.897 | 94.003 | 91.677 | 77.574 | 63.109 | 43.492 | 96.856 | 93.388 | 88.143 |
| Duplication ratio | 1.001 | 0.999 | 1.000 | 1.000 | 1.000 | 0.999 | 1.446 | 1.002 | 1.001 | 1.001 | 0.998 | 0.999 |
| # mismatches per 100 kbp | 98.57 | 105.17 | 100.53 | 94.64 | 95.03 | 87.98 | 355.60 | 187.77 | 166.86 | 95.91 | 91.84 | 78.23 |
| # indels per 100 kbp | 38.28 | 91.70 | 90.24 | 37.85 | 60.24 | 69.02 | 586.92 | 209.52 | 179.12 | 31.80 | 35.28 | 53.25 |
| Largest alignment | 13,624,614 | 3,309,419 | 3,390,099 | 15,722,595 | 5,441,037 | 4,345,189 | 362,565 | 233,146 | 113,819 | 15,697,329 | 4,511,709 | 2,320,239 |
| Total aligned length | 55,788,572 | 53,393,455 | 52,320,339 | 56,089,481 | 54,382,529 | 53,025,057 | 64,942,002 | 36,583,281 | 25,208,551 | 54,407,418 | 53,965,431 | 50,980,257 |
| NA50 | 7,025,029 | 1,153,009 | 927,337 | 8,540,456 | 2,936,488 | 1,582,761 | 72,947 | 26,014 | 15,951 | 6,805,471 | 1,961,204 | 592,850 |
| NGA50 | 7,025,029 | 907,301 | 881,469 | 8,540,456 | 2,626,025 | 1,480,643 | 79,534 | 11,037 | - | 6,805,471 | 1,557,814 | 457,946 |

**Supplementary Table 4** Computational usage of different hybrid assemblers

|  |  |  | CPU time (thread • hour) | Peak Memory (GB) |
| --- | --- | --- | --- | --- |
| Long-read coverage | 15× | AsmMix | 3.039 | 8.065 |
|  |  | WENGAN-D | 1769.590 | 838.980 |
|  |  | WENGAN-M | 441.994 | 6.863 |
|  | 75× | AsmMix | 3.184 | 7.942 |
|  |  | WENGAN-D | 2341.089 | 838.994 |
|  |  | WENGAN-M | 592.735 | 6.863 |
| Long-read length | 10 Kb | AsmMix | 3.602 | 7.579 |
|  |  | WENGAN-D | 2086.746 | 839.044 |
|  |  | WENGAN-M | 416.725 | 6.881 |
|  | 50 Kb | AsmMix | 4.193 | 7.479 |
|  |  | WENGAN-D | 1619.153 | 370.486 |
|  |  | WENGAN-M | 526.890 | 6.863 |
| Long-read accuracy | 0.75 | AsmMix | 4.538 | 8.517 |
|  |  | WENGAN-D | 2079.190 | 839.052 |
|  |  | WENGAN-M | 369.919 | 6.881 |
|  | 0.95 | AsmMix | 3.377 | 7.680 |
|  |  | WENGAN-D | 1936.343 | 370.672 |
|  |  | WENGAN-M | 665.517 | 7.543 |

**Supplementary Table 5** Benchmarking of a plant genome

|  | Haplotype-collapsed | | Haplotype1 | | | Haplotype2 | | |
| --- | --- | --- | --- | --- | --- | --- | --- | --- |
|  | Canu_ONT | Canu_PacBio | Supernova_stLFR | AsmMix_ONT | AsmMix_PacBio | Supernova_stLFR | AsmMix_ONT | AsmMix_PacBio |
| Total length (bp) | 831,238,930 | 1,204,049,054 | 879,930,553 | 844,458,961 | 1,204,137,003 | 879,552,081 | 844,454,631 | 1,204,136,491 |
| Genome fraction (%) | 44.574 | 72.362 | 68.72 | 69.727 | 72.348 | 68.709 | 69.739 | 72.349 |
| NG50 (bp) | 1,671,589 | 7,043,502 | 28,522 | 1,693,241 | 5,129,733 | 28,541 | 1,692,844 | 5,129,751 |
| NGA50 (bp) | 653 | 280,30 | - | 16,269 | 28,029 | 7,602 | 16,296 | 28,022 |
| Misassemblies/NG50 (/Mbp) | 10.643 | 12.463 | 1,636.316 | 31.271 | 17.133 | 1,622.893 | 31.344 | 17.115 |
| Local misassemblies/NG50 (/Mbp) | 15.038 | 10.755 | 1,303.590 | 28.655 | 14.760 | 1,301.391 | 28.729 | 14.760 |
| Mismatches /NG50 (/Mbp) | 1.169 | 0.339 | 85.834 | 1.437 | 0.466 | 85.803 | 1.437 | 0.466 |
| Indels /NG50 (/Mbp) | 0.672 | 0.044 | 9.457 | 0.190 | 0.060 | 9.455 | 0.190 | 0.060 |

**Supplementary Table 6.** Parameters for software tools

| **Software** | **Parameters** |
| --- | --- |
| AsmMix | default |
| stLFRdenovo | default |
| Flye | --nano-raw --genome-size 60m |
| Canu | -fast genomeSize=60m -nanopore |
| WENGAN-M (MINIA3) | -x ontraw -a M -g 60 |
| WENGAN-D (DISCOVARdenovo) | -x ontraw -a D -g 60 |
| HAST | default |
| TrioCanu | default |
| PBSIM2 (error-free) | --depth 50.0 --length-min 1000 --length-max 1000000 --difference-ratio 23:31:46 --accuracy-min 1.00 --accuracy-max 1.00 --hmm_model pbsim2/data/R95.model --length-mean 10000 --length-sd 7000 --accuracy-mean 1.00 |
| PBSIM2 (ONT) | --depth 50.0 --length-min 1000 --length-max 1000000 --difference-ratio 23:31:46 --accuracy-min 0.75 --accuracy-max 1.00 --hmm_model pbsim2/data/R95.model --length-mean 30000 --length-sd 7000 --accuracy-mean 0.85 |
| PBSIM2 (PacBio CLR) | --depth 50.0 --length-min 1000 --length-max 1000000 --difference-ratio 6:50:54 --accuracy-min 0.9 --accuracy-max 1.00 --hmm_model pbsim2/data/P6C4.model --length-mean 10000 --length-sd 7000 --accuracy-mean 0.95 |
| PBSIM2 (PacBio HiFi) | --depth 50.0 --length-min 10000 --length-max 10000 --difference-ratio 6:50:54 --accuracy-min 0.95 --accuracy-max 1.00 --hmm_model pbsim2/data/P6C4.model --length-mean 10000 --length-sd 0 --accuracy-mean 0.99 |
| QUAST | --fast -m 1000 -s |
| Merqury | default |
| BUSCO | -l primates_odb10 -m geno -sp human |
| Rtg-tools | -squash-ploidy |
| Truvari | -r 1000 -passonly |
